# HDAC Inhibitor L-Carnitine and Proteasome Inhibitor Bortezomib Synergistically Exert Anti-Tumor Activity *In Vitro* and *In Vivo*

**DOI:** 10.1371/journal.pone.0052576

**Published:** 2012-12-20

**Authors:** Hongbiao Huang, Ningning Liu, Changshan Yang, Siyan Liao, Haiping Guo, Kai Zhao, Xiaofen Li, Shouting Liu, Lixia Guan, Chunjiao Liu, Li Xu, Change Zhang, Wenbin Song, Bing Li, Ping Tang, Q. Ping Dou, Jinbao Liu

**Affiliations:** 1 Protein Modification and Degradation Lab, Department of Pathophysiology, Guangzhou Medical College, Guangdong, People’s Republic of China; 2 Department of Hematology, The People’s Hospital of Guangxi Autonomous Region, Nanning, Guangxi, People’s Republic of China; 3 Experimental Medical Research Center, Guangzhou Medical College, Guangzhou, Guangdong, People’s Republic of China; 4 Department of Hematology, The Affiliated Guangzhou first Municipal People’s Hospital of Guangzhou Medical College, Guangzhou, Guangdong, People’s Republic of China; 5 The Molecular Therapeutics Program, Barbara Ann Karmanos Cancer Institute, and Departments of Oncology, Pharmacology and Pathology, School of Medicine, Wayne State University, Detroit, Michigan, United States of America; Sun Yat-sen University Cancer Center, China

## Abstract

Combinations of proteasome inhibitors and histone deacetylases (HDAC) inhibitors appear to be the most potent to produce synergistic cytotoxicity in preclinical trials. We have recently confirmed that L-carnitine (LC) is an endogenous HDAC inhibitor. In the current study, the anti-tumor effect of LC plus proteasome inhibitor bortezomib (velcade, Vel) was investigated both in cultured hepatoma cancer cells and in Balb/c mice bearing HepG2 tumor. Cell death and cell viability were assayed by flow cytometry and MTS, respectively. Gene, mRNA expression and protein levels were detected by gene microarray, quantitative real-time PCR and Western blot, respectively. The effect of Vel on the acetylation of histone H3 associated with the p21^cip1^ gene promoter was examined by using ChIP assay and proteasome peptidase activity was detected by cell-based chymotrypsin-like (CT-like) activity assay. Here we report that (i) the combination of LC and Vel synergistically induces cytotoxicity *in vitro*; (ii) the combination also synergistically inhibits tumor growth *in vivo*; (iii) two major pathways are involved in the synergistical effects of the combinational treatment: increased p21^cip1^ expression and histone acetylation *in vitro* and *in vivo* and enhanced Vel-induced proteasome inhibition by LC. The synergistic effect of LC and Vel in cancer therapy should have great potential in the future clinical trials.

## Introduction

Inhibiting proteasome function has been demonstrated as a novel therapeutic strategy in multiple disease models like fibrosis, inflammation, ischemia-reperfusion injury and cancer [1]–[7]. Proteasome inhibitor bortezomib (velcade, Vel) has been approved by the United States Food and Drug Administration to treat multiple myeloma (MM) [8]. Other proteasome inhibitors are now under clinical trials for cancer therapy [9], [10]. Vel has achieved significant clinical benefit for multiple myeloma in clinical trials, but its effectiveness and administration have been limited by toxic side effect and development of resistance [11]–[14]. Therefore, there is still a need to search for novel combination strategies to increase its effectiveness and decrease its toxic effects. Proteasome inhibition-based combinations have been paid much attention to produce greater clinical activity [15]–[18]. Among the candidates identified in preclinical studies, combinations of proteasome inhibitors and HDAC inhibitors appear to be the most potent to produce synergistic cytotoxicity in preclinical MM models and in many other human solid and hematologic cancer cell lines and xenografts [19]–[22]. Combination therapy with Vel plus vorinostat (SAHA) in refractory MM have also been initiated in two phase I clinical trials [18]. Although the combination of proteasome inhibitor and HDAC inhibitor has a great potential to be developed as anti-cancer therapy, the involved molecular mechanism is far from being understood.

In living cells, L-carnitine (LC), a biologically active form of carnitine, is required for the transport of fatty acids from the cytosol into the mitochondria to breakdown fatty acids for ATP generation [23], [24]. Without LC, it would be impossible to burn the amount of fat necessary to produce the energy. Because of its role as a regulator in the fat-burning process, LC plays an important role in regulating weight and increasing energy levels. Therefore LC has been widely used as a “keep fit” health supplement [25], [26]. It is also known that cancer cells predominantly produce energy by a high rate of glycolysis [27], [28]. We have recently reported that LC is a HDAC inhibitor, which selectively inhibits cancer cell growth *in vivo* and *in vitro* [29].

In the current study, we investigated the synergistic effects of HDAC inhibitor LC and proteasome inhibitor Vel on cancer cell growth *in vitro* and *in vivo*, and explored the mechanism responsible for the combination-mediated cytotoxicity in cancer cells. Our findings confirmed that proteasome inhibitor and LC synergistically exert anti-cancer activity in *vitro* and *in vivo*, implying a great potential in future anti-cancer therapeutics. Our study also suggests a novel mechanism for the crosstalk between proteasome inhibition and LC-mediated protein acetylation.

## Materials and Methods

### Materials and Agents

LC was purchased from Sigma-Aldrich (St. Louis, MO, USA). Vel was purchased from Millennium Pharmaceuticals, Inc. Propidium iodide (PI) and Annexin V-FITC Apoptosis Detection Kit was purchased from Keygen Company (Nanjing, China). Fetal bovine serum (FBS) was purchased from Invitrogen Co. (Carlsbad, CA). TRIzol and Lipofecatmine 2000 were purchased from Invitrogen (Carlsbad, CA, USA). Rabbit monoclonal antibodies against Bcl-2 (50E3), caspase -3 (8G10), acetyl-H3 (Lys9) (C5B11), histone H3 (D1H2), rabbit polyclonal antibodies against nuclear poly (ADP-ribose) polymerase (PARP), acetyl-H2B (Lys5), histone H2B; mouse monoclonal antibodies against p21^cip1^ (DCS60), CHOP (L63F7), caspase-8 (1C12) and caspase-9 (C9) were all purchased from Cell Signaling (Beverly, MA). Mouse monoclonal antibodies against Bax (B-9), p27 (F-8), ubiquitin (P4D1),rabbit polyclonal antibodies against GAPDH (FL-335) and horseradish peroxidase (HRP)-labeled secondary antibodies were purchased from Santa Cruz Biotechnology Inc. (Santa Cruz, CA). Rabbit polyclonal antibody Hsp70 (SPA-812) was purchased from Stressgen Bioreagents (AnnArbor, MI). Enhanced chemiluminescence (ECL) reagents were purchased from Amersham Biosciences (Piscataway, NJ).

### Cell Viability Assay

Human hepatoma HepG2, SMMC-7721 cells were purchased from American Type Culture Collection (Manassas, VA) and grown in RPMI 1640 supplemented with 10% FBS in a humidified atmosphere with 5% CO_2_ at 37°C. The effects of drugs on the cell viability were determined by the MTS assay (CellTiter 96® AQueous One Solution Cell Proliferation assay, Promega Corporation, Madison, WI, USA). Briefly, cancer cells were cultured in 96-well plates and treated with various agents for 48h. Then treated cells were incubated with 20 µL of MTS for additional 3 h. The absorbance was measured at 490 nm with Automatic Microplate reader (Sunrise, Tecan). Three sets of experiments for each drug combinations were carried out. Cell viability was calculated by the following formula: cell viability (%) = (average absorbance of treated group - average absorbance of blank)/(average absorbance of untreated group- average absorbance of blank)] × 100%.

### Combination Index

The interaction between two compounds was quantified by determining the combination index (CI). The CI was calculated by the Chou-Talalay equation [30]. The general equation for the classic isobologram is given by: CI = (D) 1/(Dx) 1+ (D) 2/(Dx) 2. Where Dx indicates the dose of one compound alone required to produce an effect, and (D) 1 and (D) 2 are the doses of compounds 1 and 2, respectively, necessary to produce the same effect in combination. CI <0.7 indicates synergism.

### Apoptosis Assay by Flow Cytometry

Apoptosis assay was performed as previously described [31]. In brief, cultured HepG2 cells were harvested and washed with cold PBS and resuspended with the binding buffer, followed by Annexin V- FITC incubation for 15 min and PI staining for another 15 min at 4°C in dark. The stained cells were analyzed with flow cytometry within 30 min.

### Morphological Characterization of Cell Death

The morphological changes of cell death were performed as described [32]. To monitor temporal changes in the incidence of cell death in the live culture condition, HepG2 cells were seeded into 12-well plates and propidium iodide (PI) was added directly to the cell culture medium, then the cells in the culture dish were kinetically imaged with an inverted fluorescence microscope equipped with a digital camera (Axio Obsever Z1, Zeiss). Phase contrast and fluorescent images were merged.

### Western Blot Analysis

Western blot was performed as described previously [32], [33]. Briefly, an equal amount of total protein extracted from cultured cells was separated by 12% SDS-PAGE and transferred to polyvinylidene difluoride (PVDF) membranes. After the transfer is completed, the blots were blocked for one hour followed by incubation with primary antibodies and horseradish peroxidase (HRP)-conjugated appropriate secondary antibodies. The bounded secondary antibodies on the PVDF membrane were reacted to the ECL detection reagents and exposed to X-ray films (Kodak, Japan).

### DNA Microarray Assay and Analysis

DNA microarray was performed by Kangchen biotech company (Shanghai) as previously reported [29]. Briefly, HepG2 cells were exposed to various doses of LC for 24 h, or with 50 nM of Vel for 9 h and 24 h, and then a mixture of 3 three cell samples treated with each agent were collected and extracted with TRIzol agents. RNA quantity and quality were measured by NanoDrop ND-1000, and RNA integrity was assessed by standard denaturing agarose gel electrophoresis. The Human 12×135K Gene Expression Array was manufactured by Roche NimbleGen. About 5 µg total RNA of each sample (a mixture of three samples) was used for labeling and array hybridization was performed. Array scanning was performed by using the Axon GenePix 4000B microarray scanner (Molecular Devices Corporation). Scanned images (TIFF format) were then imported into NimbleScan software (version 2.5) for grid alignment and expression data analysis.

### Quantitative Real-time PCR

Quantitative real-time PCR was performed as previously reported [29]. Briefly, total RNAs were extracted from HepG2 cells with TRIzol reagent and reverse transcription of purified RNA was performed using superscript III reverse transcription according to the manufacturer’s instructions (Invitrogen). Quantification of all gene transcripts was done by quantitative PCR (qPCR) using the TaKaRa SYBR Premix Ex Taq kit with Applied Biosystems 7500 Fast Real-Time PCR system. The values of P21, P27, and Bax were shown against the value of GAPDH which was used as a control. The primer sets for amplification are listed below: p21-F: 5′GTC CAG CGA CCT TCC TCA TCCA3′; p21-R: 5′CCA TAG CCT CTA CTG CCA CCA TC3′; p27-F: 5′ACT GAG GCG GAG ACG AAG GT3′; p27-R: 5′CCT GAC AAG CCA CGC AGT AGAT3′; Bax-F: 5′CTC AGG ATG CGT CCA CCA AGA3′; Bax-R: 5′GTG TCC ACG GCG GCA ATC AT; GAPDH-F: 5′CCA GCA AGA GCA CAA GAG GAA3′; GAPDH-R: 5′GGT CTA CAT GGC AAC TGT GAGG3′.

### ChIP Assay

1×10^7^ HEPG2 cells were prepared for the ChIP assay. The ChIP protocol was performed as described previously [29], [34] by Kangchen Biotech Company (Shanghai). Anti-H3K9 antibody was used to immunoprecipitate histones. All ChIP samples were done by Realtime PCR**,** using the TaKaRa PCR Thermal Cycler and Rotor-Gene 3000 Realtime PCR. p21 and p27 primers were as follows: p21 F: 5′GCC GAA GTC AGT TCC TTG TG3′, R: 5′CGG GGT CCC CTG TTG TCT3′; p27: F:5′CTC TGA GGA CAC GCA TTT GGT3′, R:5′TGC AGG TCG CTT CCT TAT TC3′. Data are presented as fold changes calculated by each antibody ChIP value (IP/Input, the percentage of input) relative to IgG control ChIP value.

### Cell-based Chymotrypsin-like (CT-like) Activity Assay

This was performed as we previously reported [33]. Briefly, cancer cells (4,000 cells) were treated with drugs for 6 hours. The drug-treated cancer cells were then incubated with the Promega Proteasome-Glo Cell-Based Assay Reagent (Promega Bioscience, Madison, WI) for 10 minutes. The CT-like proteasome activity was detected as the relative light unit (RLU) generated from the cleaved substrate in the reagent. Luminescence generated from each reaction was detected with luminescence microplate reader (Varioskan Flash 3001, Thermo, USA).

### RNA Interference

To knock down Bax expression in HEPG2 cells, siRNA targeting human Bax were synthesized and purchased from RiboBio (Guangzhou, China). siRNA with non-specific sequences were used as siRNA negative control (NC). Different siRNAs were transfected separately into cells by using Lipofecatmine 2000 (Invitrogen) reagent and medium was replaced 6 h after transfection.

### Establishment and Treatment of HepG2 Xenografts

Male Balb/c nude mice at the age of 5 weeks (18–22 g) were purchased respectively from Guangdong Animal Center and housed in a room at constant temperature with a 12-h-light/−dark cycle. The mice consumed a commercial nonpurified diet and water ad libitum. All experimental protocols were in accordance with the Guangdong Animal Center for the ethical treatment of animals and approved by the Animal experimental Committee of Guangzhou Medical College (SCXK2008-2002). Balb/c nude mice were *s.c.* inoculated in the left armpit of each mouse with HepG2 cells (1×10^6^ cells/mouse). When the tumor size reaches 50–75 mm^3^, mice were randomly divided into four groups (8 mice/per group). Nude mice bearing HepG2 tumor were *i.p* injected with vehicle, LC (400 mg/kg, once/day except day 8), or Vel (0.5 mg/kg, once/3 days) or the combination, respectively, for 15 days. Tumors were measured and tumor volume was calculated using standard formula: Width^2^ ×Length/2. Body weight, tumor weight, tumor volume were detected and summarized.

### Statistical Methods

Mean+SD is presented where applicable. Unpaired Student’s t-test or one way ANOVA is used where appropriate for determining statistic probabilities. *P* value less than 0.05 is considered significant.

## Results

### Proteasome Inhibitor Vel and LC Synergistically Induce Cancer Cell Growth Arrest and Cell Death *in vitro*

**Figure 1:**
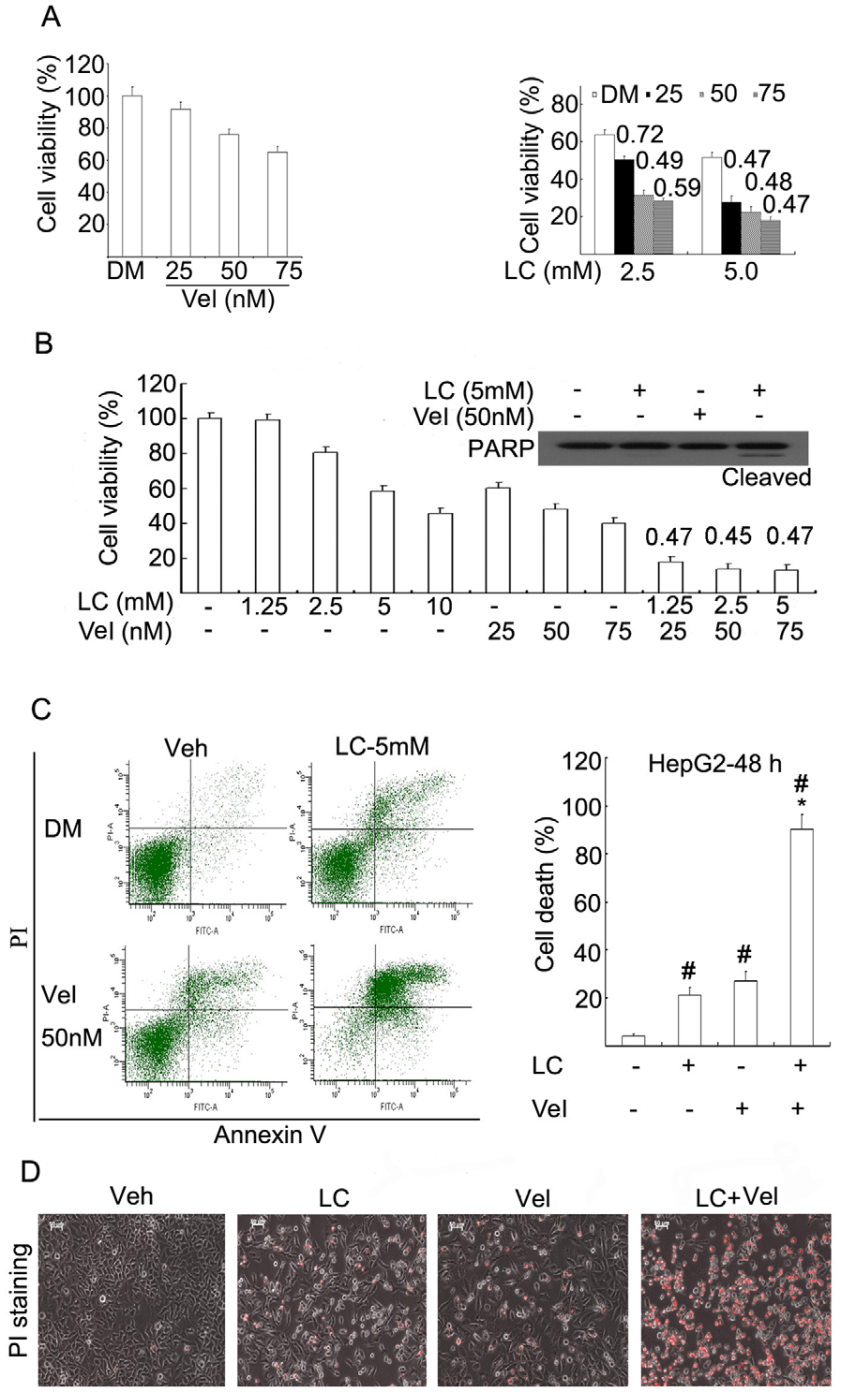
Proteasome inhibitor Vel and LC synergistically induce cancer cell proliferation arrest and cell death *in vitro*. (A) HepG2 cells were treated with various doses of Vel as indicated in the presence (2.5, 5.0 mM) or the absence of LC for 24 h, cell viability was detected by MTS assay. Mean+SD (n = 3). Combination index (CI) was shown. Vel: bortezomib; DM: DMSO; LC: L-carnitine. (B) Human hepatic SMMC-7721 cancer cells were treated with LC and/or Vel for 48 h, cell viability was detected by MTS assay and PARP cleavage was detected by Western blot. CI was shown. (C) HepG2 cells were treated with the combination of Vel (50 nM) and LC (5 mM) for 48 h and cell apoptosis was labeled with Annexin V and propidium iodide (PI), and detected by flow cytometry. Representative flow images were shown and cell death data was summarized. Mean+SD (n = 3). *P<0.05, compared with Vel or LC treatment alone; #*P*<0.05, compared with Veh control. (D) as treated in C, living cells were directly stained with PI and imaged under an inverted fluorescent miscoscope. The phase contrast and fluorescent images were taken and merged. Red indicates PI-positive. Typical images were shown.

First we investigated the effect of LC, Vel and their combination on cell proliferation in two hepatoma cancer cell lines (HepG2 and SMMC-7721). We found that Vel dose-dependently decreased cell viability in HepG2 cancer cells, consistent to previous report [35], [36], and the combination of LC (2.5, 5.0 mM) and Vel (25, 50, 75 nM) for 48 h significantly decreased cell viability with an combination index (CI) of less than 0.7 (Fig. 1A), implying a synergistic cytotoxic effect. Similar to in HepG2 cells, the combination also synergistically decreased cancer cell viability and induced PARP cleavage (an apoptosis indicator, Fig. 1B) in SMMC-7721 cells. To detect the effect of the combination on cell death, HepG2 cells were exposed to either LC (5 mM), Vel (50 nM) alone or the combination for 48 h, and cell death was detected by either Annexin-V and propidium iodide (PI) staining with flow cytometry or by PI staining under a fluorescent microscope in living cells. LC and Vel alone produced 20–30% of cell death, respectively, while the combination caused ∼90% of cell death (Fig. 1C). The morphological study in living cells also showed that LC or Vel alone induced only a few PI-positive cells (dead cells) but the combination induced high levels of PI-positive cells (Fig. 1D). These results demonstrated that the combination of LC and Vel significantly enhanced cytotoxicity in hepatoma cancer cells.

### Vel or LC Increases p21^cip1^ Expression and Accumulation of Acetylated Histones in Chromatin Associated with p21^cip1^ Gene

**Figure 2:**
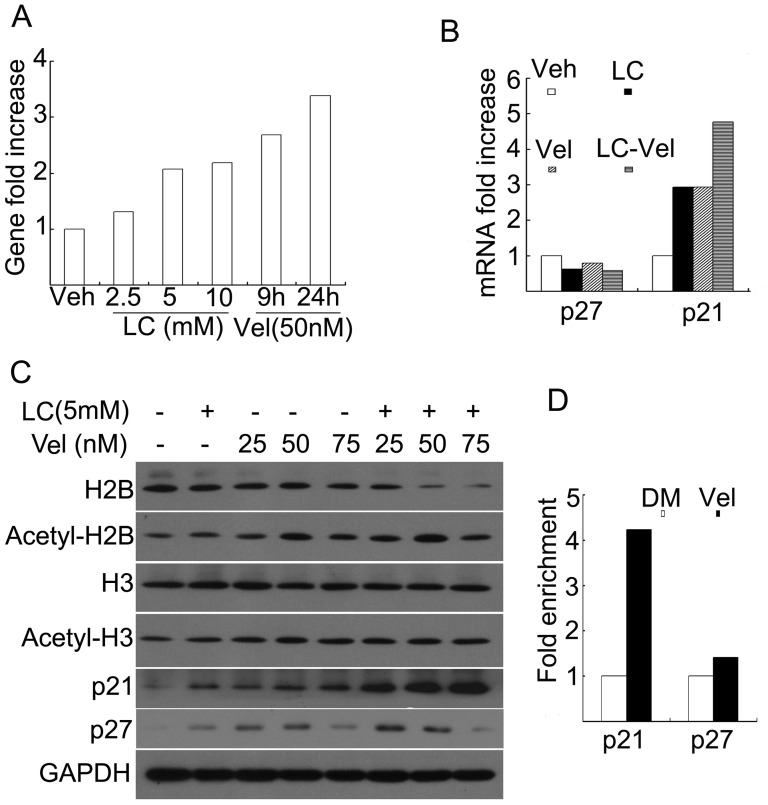
Vel and LC induces p21^cip1^ expression and histone expression. (**A**) p21^cip1^ gene expression. HepG2 cancer cells were either treated with various doses of LC (2.5, 5, 10 mM) for 24 h, or with 50 nM of Vel for 9 h and 24 h. A mixture of three cell samples were collected and DNA microarray assay was performed. Fold increase of the LC-treated *versus* control was shown. (**B**) p21^cip1^ mRNA expression. HepG2 cells were exposed to either LC (5 mM) and Vel (50 nM) or the combination for 18 h; a mixture of three cell samples were extracted for mRNA assay by real-time-PCR. Fold increases of p21^cip1^ and p27^kip1^ were shown. (**C**) p21^cip1^ protein expression and histone acetylation. HepG2 cells were treated with the combination of LC (5 mM) and various doses of Vel as indicated for 24 h, protein levels were detected by Western blot. Antibodies against p21^cip1^, p27^kip1^, histone and acetylated histones were used. GAPDH was used as a loading control. At least three repeats were performed and representative images were shown. (**D**) ChIP assay. HepG2 cells were treated with either vehicle or Vel (50 nM) for 24 h; cells were collected for ChIP assay. Fold enrichment of p21^cip1^ and p27^kip1^ promoter gene was summarized.

To determine the effect of the combination on p21^cip1^ expression, levels of p21^cip1^ gene, mRNA and protein were detected by gene microarray analysis, real-time PCR and Western blot, respectively. Consistent to previous reports [37], [38], Vel induced p21^cip1^ gene expression in HepG2 cells after treatment at 50 nM for 9 or 24 h (Fig. 2A). Further study showed that Vel or LC each alone could induce ∼3 fold increase of p21^cip1^, but not p27^kip1^ mRNA expression, while the combination induced ∼5 fold increase of p21^cip1^ mRNA expression (Fig. 2B). Similarly, p21^cip1^ protein level was also increased much more significantly by the combinational treatment than each alone (Fig. 2C). Histone acetylation after the combination treatment was then detected by Western blot. As shown in Fig. 2C, either Vel or LC treatment increased H2B and H3 acetylation, respectively, while the combination only slightly increased H2B and H3 acetylation which is possibly due to the combination-induced cell death. The effect of Vel on the acetylation of histone H3 (H3K9) associated with the p21^cip1^ gene promoter was then examined by using ChIP. The results showed that Vel, similar to LC [29], induced accumulation of acetylated histones in chromatin associated with the p21^cip1^ gene but not p27^kip1^ (Fig. 2D).

### LC Enhanced Vel-induced Proteasome Inhibition

**Figure 3:**
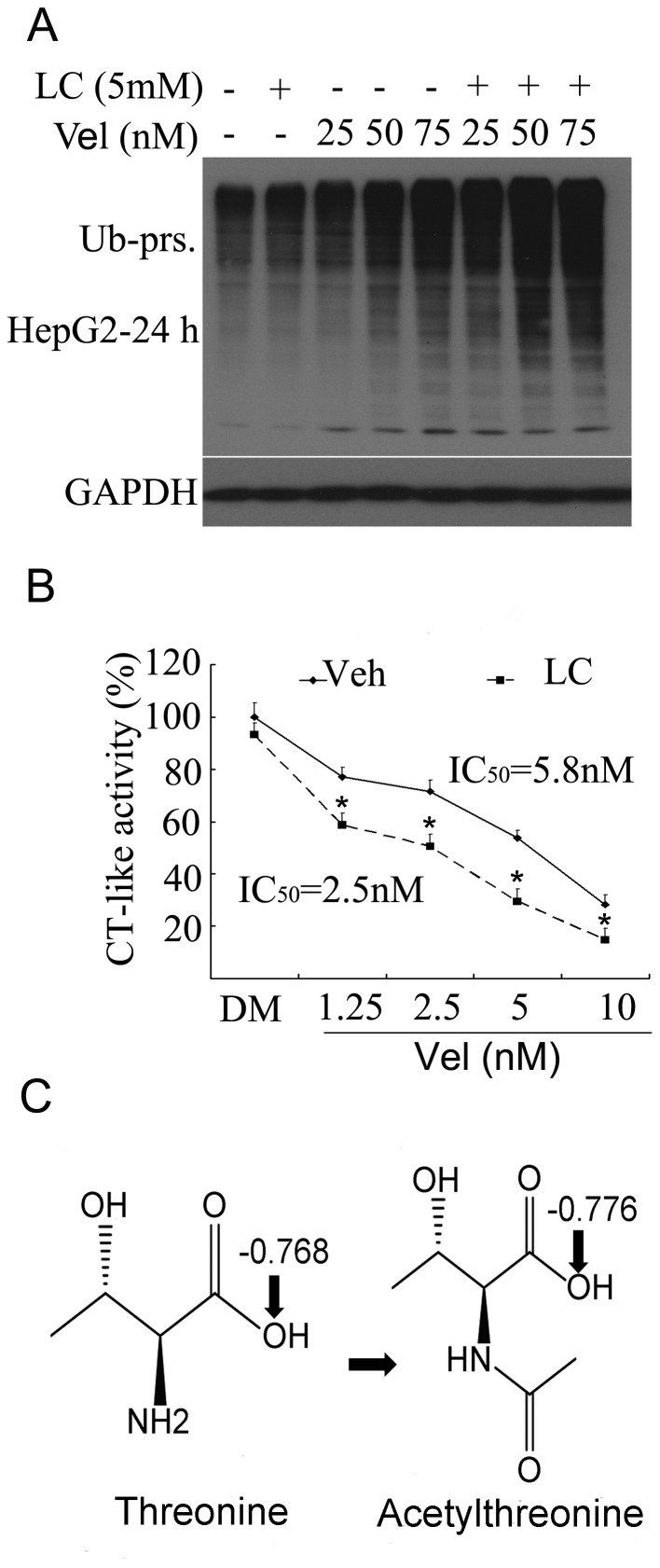
LC enhances Vel-induced proteasome inhibition. (**A**) HepG2 cells were treated with LC (5 mM) and/or Vel (25, 50, 75 nM) for 24 h, and ubiquitinated proteins were detected by Western blot. (**B**) HepG2 cells were treated with either vehicle or LC (5 mM) for 24 h, then various doses of Vel were added to the culture medium for 8 h and chymotrypsin-like (CT-like) activity *in situ* was detected. Mean+SD (n = 3). *P<0.05, compared with the control at each point. (**C**) The natural bond Orbital (NBO) charge and geometric optimization were calculated by the DFT method and the NBO charges in the hydroxyl O atom of threonine or acetylthreonine was shown.

To test whether LC, like other HDAC inhibitors, could promote Vel-induced proteasome inhibition, HepG2 cells were treated with various doses of Vel (25, 50, 75 nM) in combination with 5 mM LC for 24 h, and ubiquitinated proteins were then detected. Vel dose-dependently accumulated ubiquitinated proteins which were further enhanced by LC (Fig. 3A). To confirm this result, the CT-like activity of the proteasome β5 subunit was detected by using cell-based CT-like assay. As shown in Fig. 3B, Vel inhibited CT-like activity with an IC_50_ value of 5.8 nM, while in the presence of 5 mM LC in the medium, Vel inhibited CT-like activity with an IC_50_ value of 2.5 nM. We have found that L-carnitine increases not only histone acetylation but also acetylation of other proteins [29], and therefore we hypothesize that proteasome β5 subunit could also be acetylated. It has been reported that N^α^-acetylation of the N-terminal catalytic threonine residue in the proteasome catalytic subunits plays an important role in regulating the proteolytic activity and proteasome assembly [39], [40]. The proteasomal subunits β5, β2 and β1 in 20S catalytic core are responsible for three main proteolytic activities of the proteasome, CT-like, trypsin-like and caspase-like activities, respectively [41], [42]. A threonine residue at the N terminus (Thr1) of these subunits imparts the catalytic activity of the proteasome [43]. The atom O^γ^ of Thr1 (Thr1 O^γ^) is activated to be nucleophilic by proton shuttling from Thr1 O^γ^ to the proton acceptor Thr1 N. Compounds with electrophilic functional groups are able to react with the nucleophilic Thr1 O^γ^, causing interference of the proteasomal activity. We analyzed how the threonine residue acetylation would affect the sensitivity to Vel by using a computer model. In order to explain the interaction ability of threonine and acetylthreonine, the natural bond Orbital (NBO) charge and geometric optimization were calculated by the DFT method at the level of Becke’s three-parameter hybrid functional (B3LYP) and 6-31G (d,p) using the Gaussian 03 program. There was not an imaginary frequency appearance for all configurations at energy minima *via* the frequency calculations, which confirms that the optimized stable structures are reasonable and reliable. The calculated NBO charges disclosed that acetylation of threonine caused an decrease of the net charge for O atom of hydroxyl from −0.768 to −0.776 (Fig. 3C), indicating that the atom O^γ^ of Thr1 is activated to be more nucleophilic. This computer model result needs to be confirmed in the future experiment. These observations confirm that LC could enhance Vel-induced proteasome inhibition possibally *via* increasing acetylation of proteasome β5 subunit.

### LC and Vel Synergistically Induce Unfolded Protein Response (UPR) and Caspase Activation

**Figure 4:**
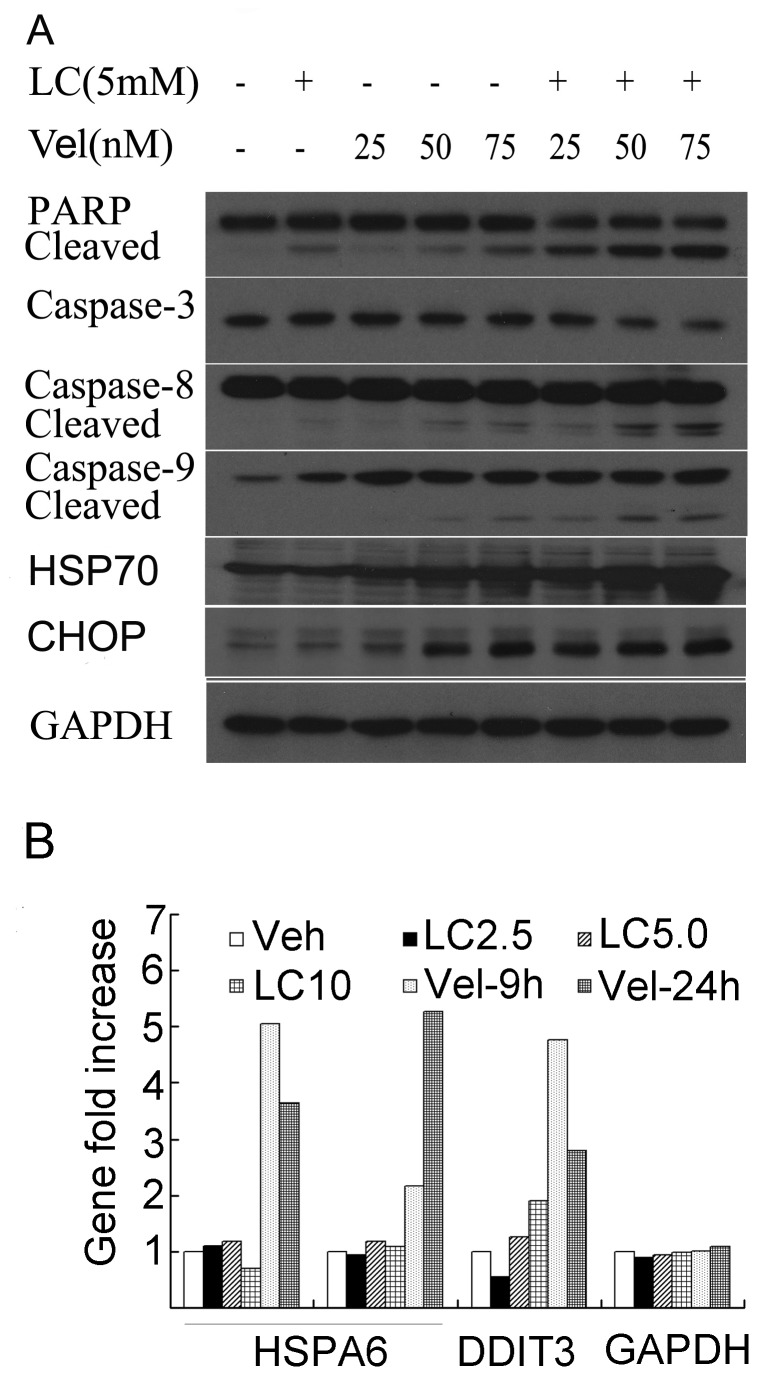
LC and Vel synergistically induces ER stress and caspase activation. (A) HepG2 cells were treated with LC (5 mM), Vel (25, 50, 75 nM) and the combination of them for 24 h. Western blot was performed for the detection of the related proteins including caspases, PARP and ER-related proteins. GAPDH was used as a loading control. (B) As treated and analyzed in Figure 2A, ER-stress-related gene expression as indicated was shown.

We further tested whether LC could promote Vel-induced UPR. As shown in Fig. 4A, in HepG2 cells, Vel alone increased the protein expression of HSP70 and CHOP, and the combination treatment greatly increased the protein expression of HSP70 and CHOP compared to Vel treatment. Further gene expression analysis in HepG2 cells after treatment with either LC (2.5, 5.0, 10 mM for 9 h) or Vel (50 nM for 9 h and 24 h) found that Vel alone markedly increased, but LC alone did not increase HSPA6 (encoding HSP70) and DDIT3 (encoding CHOP) gene expression (Fig. 4B), consistent to the changes on HSP70 and CHOP protein levels (Fig. 4A).

Next we investigated the effect of the combination on apoptosis-related proteins. It was found that various doses of Vel (25, 50, 75 nM) alone induced caspase activation and PARP cleavage, consistent to previous reports (32–34); the combination with LC (5 mM) synergistically enhanced these apoptotic changes (Fig. 4A). These results imply that the combination of these two agents strongly enhanced ER stress and caspase activation.

### LC and Vel Synergistically Induce Bax Accumulation

**Figure 5:**
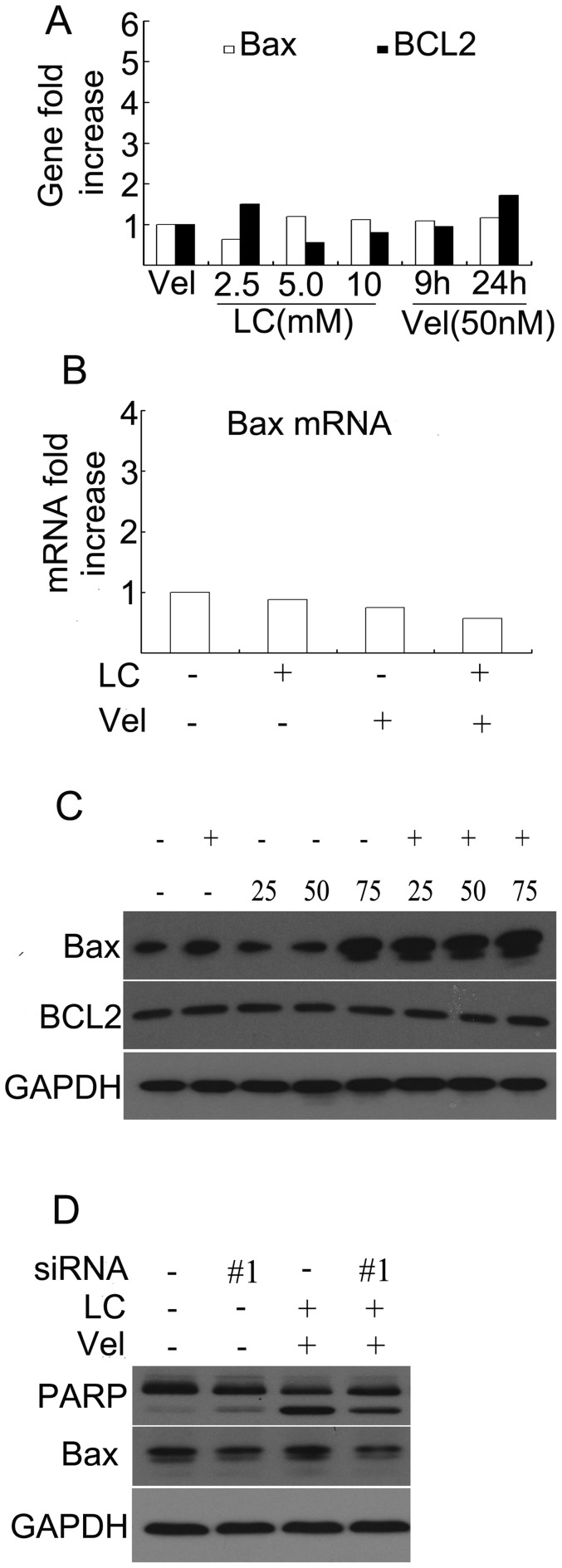
Vel and LC synergistically induces Bax accumulation and silencing Bax by siRNA reversed PARP cleavage. (**A**) As treated and analyzed in Figure 2A, Bax and Bcl-2 gene expression was shown. (**B**) As treated and analyzed in Figure 2B, Bax mRNA was detected by real-time PCR and fold increase of Bax mRNA was shown. (**C**) As treated in Figure 4A, Bax and Bcl-2 protein levels were detected. Representative images were shown. (**D**) HepG2 cells were transfected with Bax-siRNA (#1) for 48 h and then treated with the combination of LC (5 mM) and Vel (50 nM) for 24 h, Western blot was performed to detect Bax and PARP cleavage. GAPDH was used as a loading control.

To detect the effect of the combination treatment on Bax expression, levels of Bax gene, mRNA and protein expression were measured by DNA microarray, real-time PCR and Western blot, respectively. It was found that either LC or Vel alone did not affect either the gene or the mRNA level of Bax and Bcl-2 (Fig. 5A and 5B), and the combination did not affect the mRNA expression of Bax either (Fig. 5B). Protein analysis by Western blot shows that Vel at relatively high dose (75 nM) accumulated Bax accumulation and the combination dramatically enhanced the accumulation of Bax protein (Fig. 5C). These results imply that Bax increases after the combination treatment is at the post-transcriptional level and further confirm that LC enhanced Vel-induced proteasome inhibition. We then tested the important role of Bax protein in the combination-induced cell apoptosis. HepG2 cells were transfected with Bax siRNA for 48 h, and then treated with the combination of LC and Vel. It was found that #1 siRNA efficiently down-regulated Bax expression and partially inhibited the combination-induced PARP cleavage, a typical indicator of cell apoptosis (Fig. 5D). This result shows that Bax accumulation contributed to the combination-induced cell apoptosis.

### LC and Vel Combination Increases Histone Acetylation and p21^cip1^ Expression and Inhibits Cancer Growth *in vivo*

**Figure 6:**
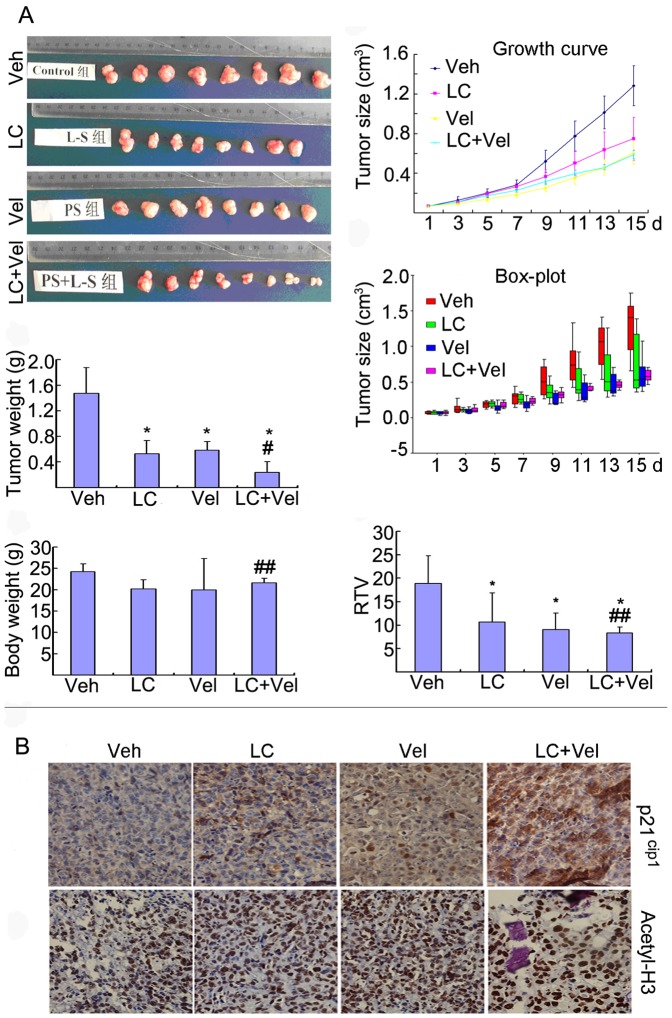
LC and Vel synergistically inhibit cancer growth and increase histone acetylation and p21^cip1^ expression *in vivo*. Nude mice bearing HepG2 tumor were *i.p* injected with vehicle or LC (400 mg/kg, *i.p.* once/day except day 8), Vel (0.75 mg/kg, *i.v.* once/3 days) alone and the combination respectively for 15 days. A, Tumor images, tumor weight, body weight, tumor growth curve including Box-plot image and the relative tumor volume in all the four groups (Vehicle-, LC-, Vel- and LC+Vel-) were shown. *P<0.05, compared with the Veh control; #*P*<0.05, compared with the treatment alone, ##*P*>0.05, compared with the treatment alone. B, p21^cip1^ and acetylated H3 proteins in tumor tissues of various groups were detected by immunochemistry and the results were shown respectively. All the immunostaining were repeated in three mouse tumor tissues and the typical images were shown.

We next observed the effects of the combination of LC and Vel on tumor growth *in vivo*. Nude mice bearing HepG2 cells were treated with LC (400 mg/kg, *i.p.* once/day except day 8), Vel (0.75 mg/kg, *i.v.* once/3 days) and the combination for 15 days. As shown in Fig. 6A, LC or Vel alone inhibited tumor growth. However, the combination further inhibited tumor growth without decreasing body weight. Similar to what observed in cultured cells (Fig. 2), LC or Vel alone moderately, and the combination strongly increased p21^cip1^ protein level in tumor tissues (Fig. 6B). Accordingly acetylated H3 protein was increased significantly in tumor tissues after the combination treatment (Fig. 6B). These results demonstrate that the combination exerts anti-tumor activity *in vivo*, associated with p21^cip1^ overexpression and protein acetylation.

## Discussion

Combination therapy of proteasome inhibitor and HDAC inhibitor has been confirmed to be promising in cancer therapeutics [19]–[22]. In the current study, we report that LC and Vel combination efficiently exerts anti-tumor effect both *in vitro* and *in vivo*. This has been confirmed by the following results. The combination (i) decreased cell viability both in hetaptic HepG2 and SMMC-7721 cancer cells; (ii) induced cancer cell death *in vitro* detected by flow cytometry, morphological observation and PARP cleavage; (iii) inhibited tumor growth *in vivo*.

Two models for the mechanism of enhancing cytotoxicity by HDAC inhibitors and proteasome inhibitors have been recently proposed [44]. One model is that HDAC inhibitors promote proteasome inhibition-induced proteotoxic stress. By blocking the proteasome, proteasome inhibitors enhance the accumulation of damaged and misfolded proteins, thus inducing downstream free radical accumulation, ER stress and caspase activation [21], [22]; the second is that proteasome inhibitors enhance HDAC inhibition. In this model, HDAC inhibitors serves as the primary cytotoxic stimulus, perhaps by promoting expression of “death genes” *via* histone acetylation [21], [22].

**Figure 7:**
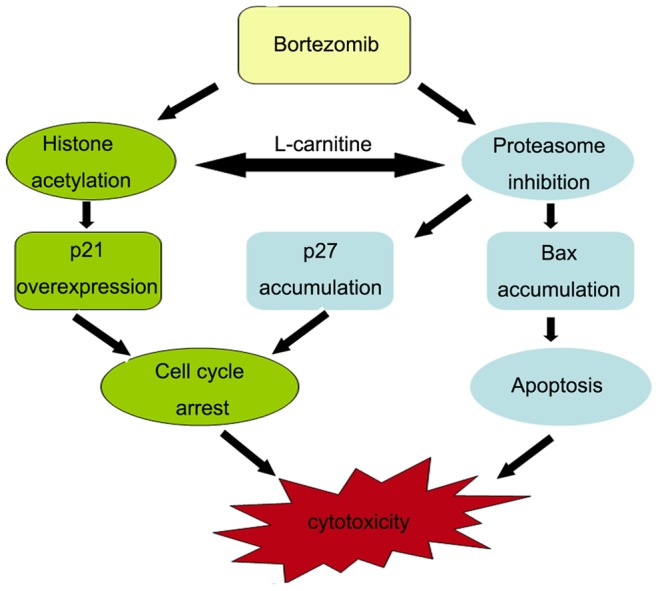
A proposed mechanism of the synergistic effect of LC and Vel on cytotoxicity. Proteasome inhibitor Vel induces proteasome inhibition and histone acetylation which is enhanced by the HDAC inhibitor LC. Proteasome inhibition enhances accumulation of Bax protein and cell cycle inhibitors p21 and p27 proteins, and histone acetylation also further induces p21 expression in cancer cells, both of which contributes to cytotoxicity mediated by the combination of LC and Vel.

Based on our findings, two pathways for the crosstalk between HDAC inhibition and proteasome inhibition have been proposed in this study (Fig. 7). One pathway is that the combination synergistically increases p21^cip1^ expression and histone acetylation *in vitro* and *in vivo*, and the second is that LC could directly enhance Vel-induced proteasome inhibition. Our results are consistent to previous reports [44].

It has been reported that HDAC inhibitors could promote proteasome inhibition-induced proteotoxic stress *via* an unknown mechanism [44]. We found that LC could (i) enhance accumulation of ubiquitinated proteins indicative of proteasome inhibition; (ii) further enhance the decrease of CT-like activity induced by Vel; (iii) induce Bax accumulation at a post-transcriptional level. These results demonstrate that LC enhanced Vel-induced proteasome inhibition. How LC sensitizes Vel-induced proteasome inhibition needs to be further investigated. Since LC as a HDAC inhibitor could induce multiple protein acetylations, this modification would affect protein degradation. On one hand, protein modification like acetylation would affect protein ubiquitination thus inhibiting protein degradation by the ubiquitin-proteasome system [39], [40]; On the other hand, the proteasome β5 subunit modification by acetylation could not be excluded.

Proteasome inhibition has been well known to induce cell death *via* multiple mechanisms including activating unfolded protein response [45]. As expected, proteasome inhibition by Vel dose-dependently induced UPR; the combination therapy enhanced this UPR and accordingly initiated caspase activation. We have reported that Bax accumulation plays an important role in proteasome inhibition-induced cell apoptosis [46], in the current study, it was confirmed that Bax plays an important role in the combination-induced cell apoptosis.

It is known that proteasome inhibitors could induce p21^cip1^ gene expression and we have also found that LC as a HDAC inhibitor could selectively induce p21^cip1^ gene expression and histone acetylation [29]. Therefore, we investigated whether these two agents could synergistically induce p21^cip1^ gene expression. Both *in vitro* and *in vivo*, p21^cip1^ expression was highly increased after the combination treatment. As reported previously, proteasome inhibitor, Vel, could increase histone acetylation by down-regulating HDAC expression [47] and therefore, we investigated the effect of the combination on histone acetylation. Even though we did not see much changes of all the HDAC gene expression (data not shown) contrary to previuos report [47], here we did find that Vel and LC combination increased histone acetylation especially in the animal tumor tissues (Fig. 6B). Like HDAC inhibitors, the accumulation of acetylated histones by either LC or Vel does not appear to be global. The GAPDH and p27^kip1^ genes are not transcriptionally activated, and there is no change in the level of acetylated histone in chromatin associated with these genes in response to LC or Vel (Fig. 2D). Even though it has been reported that Vel could increase p21^cip1^ expression [37], [38] or histone acetylation [47] respectively, this is the first time to report that Vel increases p21^cip1^ expression associated with p21^cip1^ promoter gene-related histone acetylation. In this study, it looks like that Vel-induced histone acetylation is not associated with HDAC downregulation, contrary to the previous report, which need to be investigated in the future. These results confirmed that the combination of Vel and LC synergistically and selectively induced p21^cip1^ expression associated with the accumulation of acetylated histones in chromatin associated with the p21^cip1^ gene but not p27^kip1^, which possibly contributed to cell proliferation arrest [48], [49].

Vel has been approved by FDA to treat multiple myeloma malignance [8] and also tested under clinical trial in some solid tumors [50], [51], and LC has been widely and safely used as heath supplement under many clinical conditions [26], [27]. Therefore, the synergistic effect of LC and Vel in cancer therapy will have great potential in the future clinical trials.
